# MyGeneFriends: A Social Network Linking Genes, Genetic Diseases, and Researchers

**DOI:** 10.2196/jmir.6676

**Published:** 2017-06-16

**Authors:** Alexis Allot, Kirsley Chennen, Yannis Nevers, Laetitia Poidevin, Arnaud Kress, Raymond Ripp, Julie Dawn Thompson, Olivier Poch, Odile Lecompte

**Affiliations:** ^1^ ICUBE UMR 7357 Complex Systems and Translational Bioinformatics Université de Strasbourg - CNRS - FMTS Strasbourg France

**Keywords:** health care, social media, genetic variation, hereditary disease

## Abstract

**Background:**

The constant and massive increase of biological data offers unprecedented opportunities to decipher the function and evolution of genes and their roles in human diseases. However, the multiplicity of sources and flow of data mean that efficient access to useful information and knowledge production has become a major challenge. This challenge can be addressed by taking inspiration from Web 2.0 and particularly social networks, which are at the forefront of big data exploration and human-data interaction.

**Objective:**

MyGeneFriends is a Web platform inspired by social networks, devoted to genetic disease analysis, and organized around three types of proactive agents: genes, humans, and genetic diseases. The aim of this study was to improve exploration and exploitation of biological, postgenomic era big data.

**Methods:**

MyGeneFriends leverages conventions popularized by top social networks (Facebook, LinkedIn, etc), such as networks of friends, profile pages, friendship recommendations, affinity scores, news feeds, content recommendation, and data visualization.

**Results:**

MyGeneFriends provides simple and intuitive interactions with data through evaluation and visualization of connections (friendships) between genes, humans, and diseases. The platform suggests new friends and publications and allows agents to follow the activity of their friends. It dynamically personalizes information depending on the user’s specific interests and provides an efficient way to share information with collaborators. Furthermore, the user’s behavior itself generates new information that constitutes an added value integrated in the network, which can be used to discover new connections between biological agents.

**Conclusions:**

We have developed MyGeneFriends, a Web platform leveraging conventions from popular social networks to redefine the relationship between humans and biological big data and improve human processing of biomedical data. MyGeneFriends is available at lbgi.fr/mygenefriends.

## Introduction

### Social and Scientific Contexts

Web 2.0 and, particularly, social networks (Facebook, Google+, and LinkedIn), interconnect billions of users and manage terabytes of dynamic data flow [1]. They are at the forefront of the interactions and cooperation between humans and big data, and as such, they have established or popularized new conventions. A central concept in these innovations is the notion of an agent, representing an autonomous and active network member with various prerogatives. Notably, an agent can (1) add new information, via micro-blogging for example; (2) spread information through the network via sharing [2]; (3) evaluate information with like, dislike, or vote reactions; (4) partition information using privacy settings; or (5) annotate information with comments. Agents play an active role in the evolution of the network structure by creating nodes (agent profile pages) and bidirectional (friendship) or unidirectional (follower) links between agents. They also partition agents into groups and connect agents to unstructured information (tagging). These actions are processed by specialized tools embedded in the network to create valuable feedback in the form of filtered and personalized information such as friendship suggestions, affinity scores between people, news feeds, targeted advertisements [3], merchandise suggestions [4], or real-time world observations [5].

The field of biology is evolving and adapting at a tremendous rate in response to the widespread use of high throughput methods and the rise of personal genomics [6]. For the end user of biological data, the paradigm shift initiated by the emergence of this big data [7] has led to important changes in the research landscape [8]. To keep up with the huge volumes of data and information, users need to easily and intuitively access, communicate, and network with useful information of personal interest. Therefore, data storage platforms and workflow infrastructures must evolve to integrate Web 2.0 and social network conventions.

### Bioinformatics in the Web 2.0 Era

In this context, several major bioinformatics resources have introduced tools for personalized data flow management. The Online Mendelian Inheritance in Man (OMIM) [9] resource now proposes MIMmatch [10], a service allowing users to receive email notifications when entries for their favorite genes or diseases have changed. MyNCBI [11] retains user preferences to provide customized services for NCBI databases, whereas the Uniprot [12] website has been updated to allow users to select only categories of information they are interested in, to mask large-scale publications, and to use a basket to store proteins of interest. Similar efforts toward more efficient and personalized information management are also emerging in the exploitation of the increasing publication flow. Bibsonomy [13] allows a researcher to collect and manage publications and collaborate with colleagues, whereas PubChase and ReadCube recommend new publications depending on the content of an existing library. BioTextQuest+ [14] provides an interactive exploration platform for PubMed [15] and OMIM, and facilitates knowledge extraction by document clustering and bioentity recognition. GoPubMed [16] proposes pertinent publication searches by using background knowledge in the form of ontologies (gene ontology [GO], Medical Subject Headings [MeSH], etc) that take into account the user’s keywords, but also synonyms and child concepts, whereas DeepQA4PA [17] returns GO concepts associated with publications related to a specific question. After identifying a gene or list of genes of interest, GeneMania [18] and GenesLikeMe [19] identify and score related genes that may also interest the user based on ontologies, disorders, compounds, phenotypes, expression levels, domains, sequences, and other data.

Important efforts have also been devoted to contextualizing entities by connecting them to their network. For instance, FACTA+ [20], Pubtator [21], or PAML-IST [22] return publications and their links to various biological entities such as compounds, drugs, enzymes, genes, diseases, symptoms, mutations, species, and others. EuropePMC [23] adds connections to GO and experimental factor ontology (EFO), and iHOP [24] highlights the most recent publications linked to a protein. Interaction with this complex data has been facilitated by the progressive democratization of visualization techniques. For instance, Javascript libraries like BioJS [25] provide reusable components for visualization of biological information (3D structures, phylogenetic trees, proteomes, pathways, and multiple sequence alignments), contributed by users and stored in a registry. Visualization techniques facilitate understanding of information updates, clarify links between entities and groups of entities, and highlight metadata information such as data sources, confidence estimates, and so on. For example, the ExAC browser [26] provides clear visualization of variations in a gene, the Semantic Body Browser [27] shows gene expression in a human and a mouse with a heat map on a schematized body, and NetGestalt [28] introduces 1-dimensional visualization of network modules to facilitate network comparisons.

Conversely, other tools aim to extract relationships between entities. For example, Chilibot [29] searches interaction (stimulation, inhibition, etc) or parallel (studied together, coexistence, homology, etc) relationships between user-submitted genes or proteins. EvexDB [30] extracts specific events: regulatory control, coregulation, or binding to a given gene.

Finally, some bioinformatics resources have introduced specific collaborative and social components, with wiki-inspired approaches like Proteopedia [31] or WikiGene [32], collaborative sequence annotations such as WebApollo [33], or voting for medical relevance and scientific evidence of variations with GeneTalk [34]. Recent initiatives such as Coremine or MAGI [35] combine these trends. Coremine allows exploration of various biomedical concepts and connections between them, addition of private or public comments, alerts on new articles or connections, and bookmarking. MAGI combines public and private cancer genomics datasets with sharing and collaborative annotation features as well as with interactive visualizations of variants, gene expression, and protein-protein interactions.

### MyGeneFriends

Building on these advances, we have developed MyGeneFriends, a Web platform inspired by social networks, to redefine and enhance the relationship between humans and biological big data. By leveraging and combining conventions and practices arising from popular social networks, it provides more intuitive interactions with biological data and simplifies access to complex information by organizing it around three agents: genetic diseases, genes, and humans. This allows MyGeneFriends to be used not only by researchers and clinicians but also by the public, including empowered patients.

We focused on human genetic diseases (closely connected to genes and human users), as they represent major clinical challenges and provide a simplified context to shed light on major common diseases. MyGeneFriends allows retrieval, management, contextualization, and annotation of information related to genes (expression, localization, and so on), genetic diseases (phenotypes, variations, and so on), and humans (interests, publications, and so on). The platform leverages user behavior and networking to personalize data visualization and the flood of information for each human user’s needs, and allows project-oriented collaborations. Publication and friendship suggestions facilitate the identification of new relevant genes and diseases. Finally, we capitalize on the global social network to extract additional knowledge. MyGeneFriends was used during its development by members of our laboratory that provided continuous feedback. Additional feedback was collected from clinicians and researchers of the Medical Genetics Laboratory of Strasbourg and from colleagues from other laboratories that was particularly useful for improving the visualization of variations linked to a disease.

The aim of this paper was to introduce readers to MyGeneFriends and describe how practices from social networks can be applied to improve access to biological data.

## Methods

### Platform Architecture

The MyGeneFriends platform integrates multiple services (Multimedia Appendix 1) to extract and integrate large amounts of heterogeneous data. The data are stored and managed in a Postgres database, with a backup copy produced daily and stored on an external server. Elasticsearch [36] is used for powerful, complex, and fast plain text queries of publications and is synchronized daily with the MyGeneFriends database. The website is based on a stateless framework (Play framework) that includes many useful features such as error handling, build-in support for Json, WebServices, WebSockets, CoffeeScript, EBean object-relational mapper (ORM), localization, logging, and WebJars. The Play framework ensures easy horizontal scaling and scalability for increasing website traffic.

To execute local scripts and programs, a Web service has been developed using the Flask framework, which is called by MyGeneFriends using REST requests to run analysis or integration tasks. Data integration scripts are written in python, using peewee as the ORM.

### Data Sources

Gene-related data including gene symbol, short description, type, and protein sequence are mainly obtained from the Ensembl [37] database. UCSC provides simple access to RefSeq [38] annotations for transcripts. To map gene identifiers between Ensembl and NCBI, we combine mappings performed by Ensembl and NCBI, together with gene symbol mapping, and extract one-to-one relationships. Gene expression data are obtained from the Human Genome Atlas microarray data [39] available in the gene expression omnibus (GEO) [40] database and validated using in-house statistical methods. In addition, relative signal intensities are calculated for heat map visualization using log signal intensities normalized in the range (0-1). Cellular localization of gene products is based on cellular component terms from GO [41]. Phylogenetic distributions for human genes and 100 eukaryotic species are retrieved from the OrthoInspector database [42] and used to categorize genes according to their evolutionary profile.

The relationships between genes and publications are defined using the gene2pubmed file from the NCBI. Publication abstracts are downloaded from Pubmed and integrated in the MyGeneFriends database. The python natural language toolkit (NLTK) [43] library is used to extract keywords from textual data linked to genes and diseases. It tokenizes the text into phrases and words, stems words in order to retrieve a canonical form, and filters words on the basis of the NCBI list of stop words (words that occur frequently in texts but are not informative) and in-house filters for word size, numbers, and special characters. Then, we take advantage of the gensim [44] library to calculate the Inverse Document Frequency (IDF) of the keywords and the TF*IDF (Term Frequency * Inverse Document Frequency). The IDF is used as a specificity score, and the TF*IDF is used to weight the relationship between a keyword and a gene or disease.

The main disease-related data are obtained from OMIM and Orphanet. In order to take into account differences in disease definitions from different data sources and propose a unified view of the current disease knowledge, an integration process was developed with two simple rules (Multimedia Appendix 2). After integration, diseases are linked to phenotypes using human phenotype ontology (HPO) [45] data files (hp.obo and phenotype_annotations.tab) containing phenotypes and phenotype-disease relationships. Variations and variation-disease relationships are extracted from the curated set provided by ClinVar [46] in the variant call format (VCF) file (limited to records with an rs# identifier). Each line is parsed and a variant entry is integrated into MyGeneFriends as a couple of genomic position and allele, allowing precise definition of the relationships between diseases and mutations. Variant effect predictor (VEP) [47] is used to link variations retrieved from ClinVar to Ensembl transcripts and to estimate their effect. The effects are then automatically classified into more general categories using the sequence ontology [48] data.

### Data Flow Management

The data flow management involves the integration of data from diverse sources (databases, FTP servers, and local files) into the MyGeneFriends database. After cleaning and parsing mined data, additional analyses are automatically processed, such as keyword extraction from biological text or generation of links between variants and transcripts (mentioned previously). Then, MyGeneFriends compares remote and local data to generate news events. One or more fields from each item is used as a unique identifier. If a remote item has an identifier (one or several selected fields from an item) that is absent from the local database, it is considered to be a “new“ event. If a local item has an identifier that is not present in the remote source, it is considered to be a “delete” event. If the identifier is present in both remote and local sources, the items are compared field by field to generate “update” events. Once these events are generated, the local database is synchronized with the remote source.

Finally, the way the news item is presented to the user depends on the biological context of the considered element. When an agent is linked to a publication that is not available in the MyGeneFriends database, the publication is downloaded and made accessible directly from the news panel. When a sequence is updated, a sequence alignment is generated using ClustalW [49]. When a textual information changes, such as the description of a disease, the google-diff [50] python library is used to compare both versions of the text and highlight the differences.

### Data Display as Word Clouds

Word cloud representations are used in the visualization panel of an agent to display the cellular localization of the protein encoded by a gene and the phenotypes associated with a disease. Specific terms are considered as more informative and emphasized in the word cloud. The specificity of a term (cellular component in GO and phenotype in HPO) describing an agent is estimated using the information content (IC) metric [51]. The IC is defined as the negative natural logarithm of the probability of a term t:

IC (t)=−logP (t),

where P (t) is based on the frequency of the term in the considered ontology.

The specificity is then defined as the IC normalized in the range (0-1), where 0 corresponds to the minimal font size and 1 to the maximal font size during word cloud rendering.

### Friendships

The MyGeneFriends network is based on friendships between agents. Human friendships are defined by users, whereas gene-gene, disease-disease, and gene-disease links are automatically built from external sources (search tool for recurring instances of neighboring genes, STRING [52]; and HPO) or inferred from the MyGeneFriends network (Error: Reference source not found). STRING global scores (higher than 0.7, corresponding to “high confidence” in STRING) are used as a metric of friendship between genes based on protein-protein interaction data. Causative genes mined by HPO from OMIM and Orphanet are exploited to link genes and diseases.

In addition to these external sources, MyGeneFriends establish links based on common properties. Diseases sharing phenotypes are related to each other with a score defined as the sum of specificity scores of phenotypes common to both diseases, divided by the sum of specificity scores of all phenotypes related to both diseases. Similarly, genes sharing GO [41] terms are connected according to two different metrics. The first metric (“GO simple”) is based on the number of shared GO terms between 2 genes, whereas the second corresponds to the functional semantic similarity (FSS) [51]. Genes and diseases related to the same variant(s) are also linked. Moreover, genes are evolutionarily linked when applicable, on the basis of the Jaccard distance calculated between in-house phylogenetic profiles produced by OrthoInspector [42].

Finally, nonhuman agents can become friends based on social connections emerging from the network itself: genes sharing human or disease friends are connected, as well as diseases with common human or gene friends.

### Suggestions and Affinity Score

To suggest new gene or disease friends or new publications to a user, MyGeneFriends relies on the content of the user’s active Topic. For friendship suggestions, each nonhuman agent from MyGeneFriends (a_c_) is scored relative to the user’s active Topic and the top 10 candidates are suggested as new friends. The score of an agent (gene or disease) given a Topic is the sum of scores (S) between this agent and all agents of the same type in the active Topic (a_t_):

score(a_c_) = Ʃ^N^_t=0_ S(a_t_, a_c_)To score genes, we use the global STRING score, whereas the score between two diseases d1 and d2 is calculated using the Information Content (IC) of the related phenotypes (P) as:

Score(d1, d2) = (Ʃ *IC* (*P* ∈d1∩d2))/ (Ʃ *IC* (*P* ∈d1∪d2))

In addition, we provide an affinity score (a_aff_) reflecting the proximity between an agent and the content of the user Topic and thus, the relevance of befriending this agent. It is displayed on the gene and disease profile pages when the agent can be related to the content of the Topic. The affinity score is defined as:

a_aff_ = a_c_ /max ac X 100

To suggest pertinent publications, MyGeneFriends uses keywords associated with the active Topic. These keywords have been either added manually by the user or automatically inferred (see formula below). The keywords are weighted and used to query the Elasticsearch server to retrieve pertinent publications. For Elasticsearch, weights between 0 and 1 reduce the relevance of a term, and weights higher than 1 increase it. Therefore, the weight for each keyword given the content of the Topic (k_t_) is defined as:

weight(k_t_) = 1 + Ʃ^N^_t=0_ ka_t_ + k_h_,

where ka_t_ is the score describing the relationship between the keyword and an agent from the Topic, and k_h_ is a factor applied if the user has explicitly added this keyword to the Topic.

## Results

### Overview of the Platform

MyGeneFriends is a new social network leveraging conventions from Web 2.0 and interconnecting three kinds of autonomous and active agents: human genes, humans, and genetic diseases. All genetic disorders including malformations, groups of phenotypes, etc are included in the network, as well as all types of human genes (coding and noncoding) in agreement with the growing evidence concerning the importance of noncoding genes in biological processes and diseases [53-55].

All agent-related data is accessible via standardized profile pages. Daily data mining and integration processes have been developed to maintain the “nonhuman” agents (more than 63,000 human genes and 14,000 genetic diseases) up to date and generate a news flow (more than 1 million news items were created in the last year) by exploiting public (Ensembl [37], NCBI, Uniprot [12], HPO [45], OMIM [10], Orphanet [56], OrthoInspector [42], etc) and in-house data resources. All data retrieved or processed by MyGeneFriends and related to genes and diseases are “public,” whereas data submitted by humans are “private” (visible only by the owner) by default, unless the human decides to make it “protected” (visible by owner and selected collaborators) or “public” (visible to anyone).

**Figure 1:**
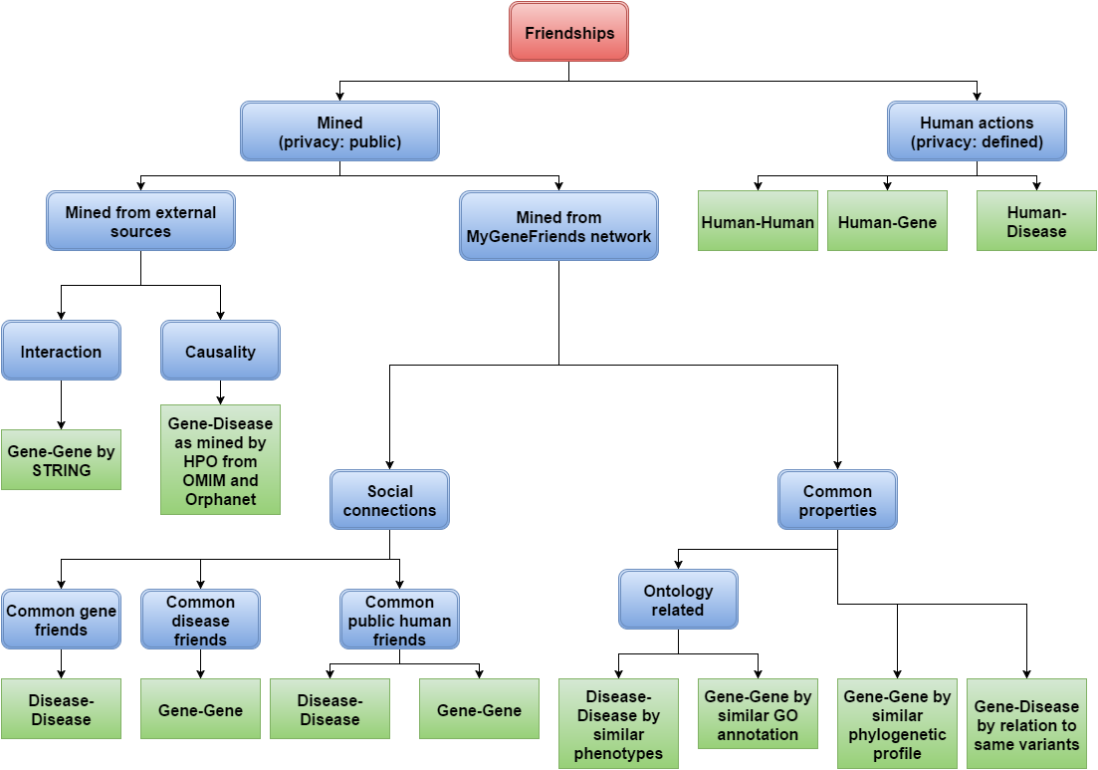
Ontology of friendships between agents in MyGeneFriends. Agents are linked by numerous friendships (corresponding to green boxes) of different kinds (blue boxes). First, we separate decision-driven friendships (agent actions) from naturally occurring friendships (mined). Then, we split natural friendships into those due to direct contact between agents, and those influenced by an external factor. This external factor mimics the human tendency of befriending people with the same interests (represented here as phenotypes, annotations, variants, and phylogenetic distributions) or common friends (genes, humans, and diseases).

The MyGeneFriends network arises from several millions of connections (called “friendships”) between agents, resulting from automated dynamic data mining processes combined with human actions (Figure 1). Assessment of gene-gene, gene-disease, and disease-disease connections (nonhuman friendships) are based on automated mining of bibliographic, evolutionary, functional, phenotypic, or social data. Human friendships with genes, diseases, and other humans are defined by the user through gene targeting, definition of research interests (Topics), or user targeting (groups). Human friendships with genes or diseases can be private, protected, or public, although they are public by default to encourage networking. This data privacy management [57] is crucial to keep essential data private, while being open enough to “attract” new information and collaborators.

By exploiting human actions, MyGeneFriends can automatically (1) personalize information and visualization by highlighting and filtering pertinent data, (2) suggest new publications and friends (gene or disease), and (3) provide subnetworks for collaborations on defined research interests.

### Agent Profiles

**Figure 2:**
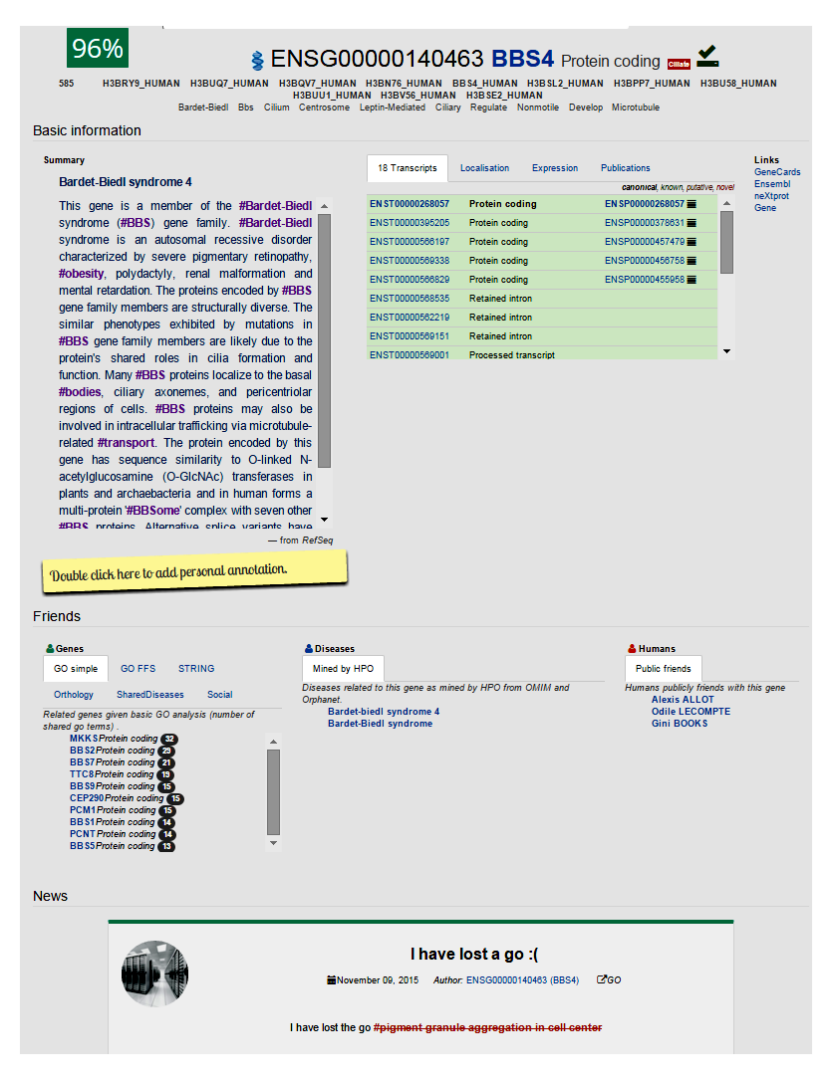
Representative profile page of a MyGeneFriends agent (here the gene BBS4). Four distinct sections are shown. The “header” section briefly introduces the agent, displays a list of synonyms, and allows friendship management. It shows the affinity score (here 96%) estimating how interesting this agent could be for the user. The “basic Information” section shows more detailed information about the agent: a description, different visualizations describing the agent, links to external sources, and a personal annotation from the user. The “friends” section allows navigation through the “friends of friends” network by displaying public friends of the agent, grouped according to their type. Finally, the “news” section displays all the news related to the agent.

Each agent in MyGeneFriends has a profile (Figure 2) that provides a unified architecture and organization to ensure intuitive navigation through the network and access to relevant and personalized information about agents. These profiles contain 4 major sections: “header,” “basic information,” “friends,” and “news.”

**Figure 3:**
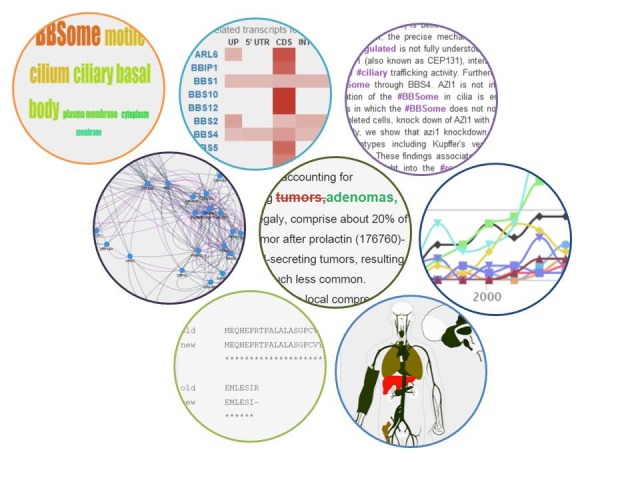
MyGeneFriends uses various visualization techniques to optimize the display of biological information: (1) word clouds highlight the most specific ontology terms, (2) barcodes offer a synaptic and interactive view of the density of variations related to regions of a gene or effect on a protein, (3) highlighting words in text identifies the most pertinent paragraphs for a given human user, (4) networks of friends help to understand the connections between agents and identify groups of highly connected agents, (5) colors highlight modifications in textual information related to agents, (6) timelines show the evolution of the popularity of gene collaborators in a Topic, (7) pairwise alignments identify the differences between two versions of protein sequences, and (8) heat maps on schemas of the human body, brain, and fetus allow easy analysis of the expression pattern of a given gene.

The top agent-related keywords are displayed in the header to briefly introduce the agent, whereas the summary in the “basic information” section provides a more detailed description. Humans can expand the official description of a nonhuman agent by adding personal annotations or unpublished results that can then be accessed at any time and shared with collaborators (Figure 2). Exploration panels give access to the most important information using visualization techniques (see Figure 3) to highlight specific information for genes and diseases as described below. The “friends” section of the profile displays links to public friends (genes, diseases, or humans) of the agent, allowing further networking with potentially interesting agents. Finally, the news feed is an intuitive way to track changes in information related to an agent.

To personalize the profile view, the keywords inferred to be important for the viewer are highlighted in the agent description. For example, if the user is friends with cilia-related genes, the word cilium is highlighted in the description of the other agents (human, gene, or disease). Moreover, if a nonhuman agent is related to the user’s current collaborators, an affinity score is shown, inviting the user to befriend this agent. If the agent already collaborates with the user, the score reflects how close it is to other collaborators.

### Gene Profile

Gene profiles use RefSeq summaries to describe agents and connect these agents to external resources via links to Ensembl, GeneCards, NCBI, and neXtprot websites.

Exploration panels display the most important aspects of the gene. The first panel presents gene transcripts with their properties: sequence, type (protein coding, miRNA, etc), reliability (known, putative, and novel), and corresponding protein sequence, if any. The second panel shows the subcellular localization(s) of the encoded proteins, defined by the GO cellular component ontology, as a word cloud (Figure 3). The third panel shows the gene expression for protein coding genes as a heat map in more than 40 tissues, through an interactive schematic view of the human body (male and female), brain, and foetus (Figure 3). Pan and zoom capabilities (jquery.panzoom.js) allow users to navigate through the schematic view and visualize even the smallest tissues. Additional information such as tissue description and probe set signal intensities are available. In addition to the visualization of gene expression, the “Expression filter” tool allows users to find genes of interest based on their expression or absence of expression in a defined set of tissues. Publications associated with the gene are displayed with their abstract and can be liked, disliked, or marked as valuable. The number of all genes related to the publication, as well as the count of likes and dislikes, help to estimate the relevance of the publication for the considered gene. Moreover, genes related to a publication can be visualized as an interactive graph, allowing further networking and identification of additional genes of interest.

### Genetic Disease Profile

Diseases are extracted from the OMIM (all entries except explicit genes) and Orphanet (all entries, including groups of phenotypes) databases. The preference of exhaustivity over specificity is motivated by the inherent difficulty in defining a disease. We use expert created links between Orphanet and OMIM entries (displayed on Orphanet entries) as the main data source to merge diseases. When a disease is not linked to any other, or when a clear one-to-one mapping can be made between an Orphanet and an OMIM entry, the entries from both databases are fused into a single one (see Multimedia Appendix 2). Once this process is complete, we use the remaining one-to-many connections (eg, one entry for “Bardet-Biedl syndrome” in Orphanet corresponds to multiple entries in OMIM for each Bardet Biedl syndrome subtype) to create groups of highly connected diseases, which we call “metadiseases.”

Two main features have been selected to characterize a disease on the disease profile panel: (1) variations explaining the causes of a disease, and (2) phenotypes describing its consequences. Phenotypes are represented by a word cloud highlighting rare HPO phenotypes associated with the disease. The description of variants is generated by the integration of more than 100,000 ClinVar [46] curated variations (single-nucleotide variants and small insertions and deletions) directly linked to diseases.

As the effects of the variants can differ per considered transcript, MyGeneFriends uses the Ensembl VEP [47] script to create more than a million links between variants and Ensembl transcripts stored in the MyGeneFriends database. To describe the complex relationships between variants, transcripts, and disease-causing genes, we have developed three synoptic and interactive views with variants grouped per affected gene. With this synthetic barcode representation (Figure 3), the human user has a rapid overview of the characteristics of the known variants associated with the disease and can easily identify variants exhibiting specific features, for instance, synonymous variants affecting a splicing region. The third view focuses on variants differentially affecting protein coding transcripts (Multimedia Appendix 3). Such variants can generate a mix of affected and unaffected proteins depending on the tissue or developmental stage and often result in puzzling phenotypes.

Metadiseases have special profile pages on MyGeneFriends, summarizing the main properties of nested diseases, displaying nested diseases as a network, and highlighting the most representative gene friends and phenotypes of the concerned diseases. To date, MyGeneFriends has information on 725 metadiseases, representing 3418 diseases.

### Human Profile

The third agent in MyGeneFriends is the human user, who must register on the website (registration is free, and a demo account is available for testing purposes). The user’s profile page contains information provided by the owner: his affiliation, geographic localization, a list of authored publications, and a short description. Even if no description is provided, MyGeneFriends introduces the human to other users by automatically extracting best scored keywords associated with public gene and disease friends of the human and displaying them on his profile.

The private view of the profile page allows humans to create and manage groups of collaborators related to research projects, called Topics. All Topics owned by the user are shown in the “My Topics” section. The Topic selected as “active” is used for personalization and suggestion processes. A second section called “My collaborations” allows the user to monitor Topics from the other users with whom he collaborates.

### Friendships and Networking

Friendships are an essential concept in MyGeneFriends, since on the one hand, they allow networking through friends and evaluation of the relatedness of 2 agents, and on the other hand, they are used to suggest interesting agents as new friends. Some friendships are automatically generated based on data mining, whereas others result from human activity. Friendships offer different and complementary points of view on the close environment of an agent in terms of protein interactions, function and localization, implication in research projects or diseases, and many others (Figure 1).

**Figure 4:**
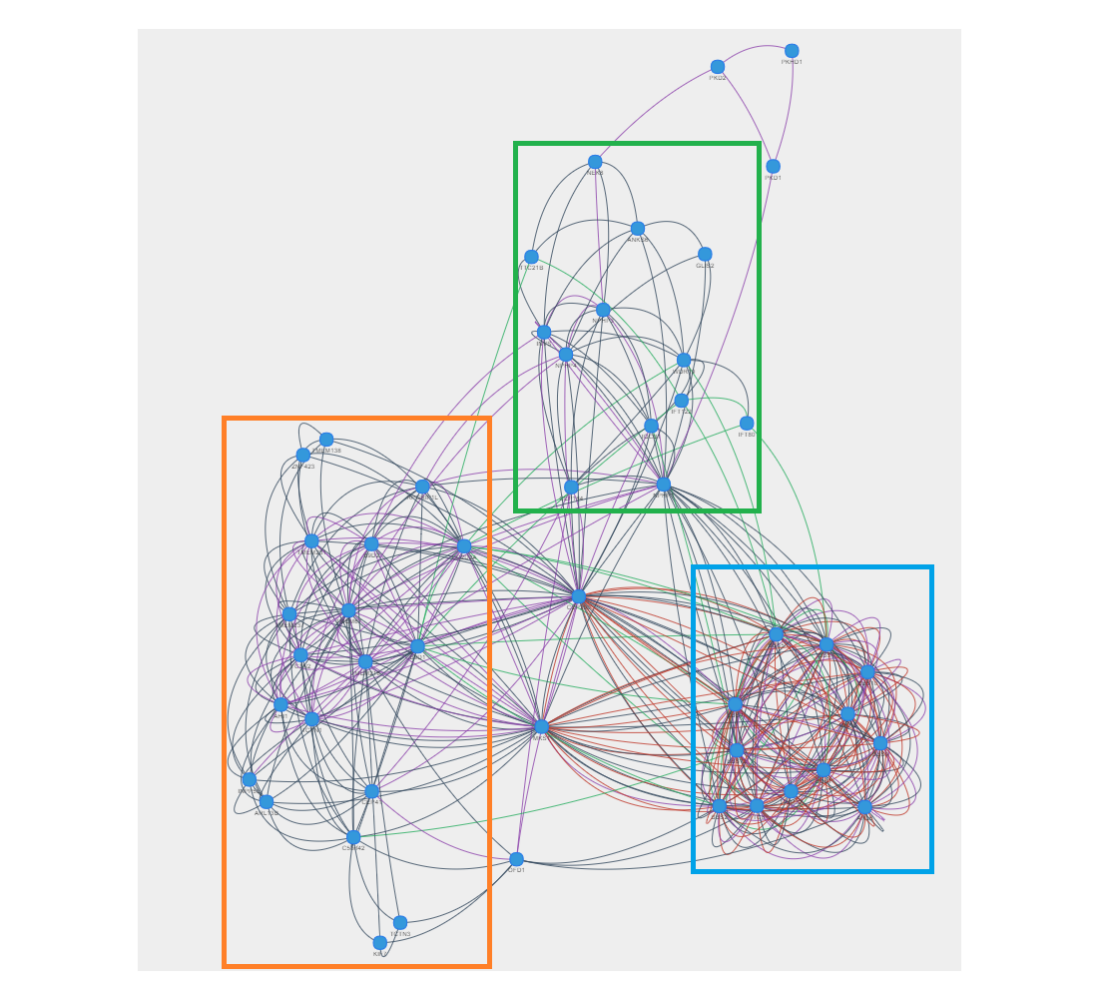
Dynamic network visualization of relationships between actors. Network of 52 genes related to Congenital Hepatic Fibrosis (CHF), a developmental disorder most frequently associated with ciliopathies. Red links represent shared public human friends, grey links represent shared diseases, violet links represent STRING relationships, and green links represent similar evolutionary profiles. Each link type can be removed or added to the network in real time. Moreover, in the dynamic network view provided by MyGeneFriends, highly connected genes are clustered automatically to form subgroups. In this example, 3 main subnetworks (highlighted by rectangles) emerge corresponding to genes associated with a distinct ciliopathy: Bardet-Biedl Syndrome (blue rectangle), Joubert and Meckel syndromes (orange rectangle), and Senior-Loken syndrome and nephronophthisis (green rectangle).

Exploitation of the friendship network in MyGeneFriends leverages mined and user-created connections to discover highly connected clusters. Interactive graph views with repulsion physics (using the vis.js library) allow intuitive visualization of friendships within a group of agents (genes from a publication, diseases from a metadisease, or agents associated with a Topic), leading to selection and observation of different types of friendships (common friends, common features, cooccurrence, and so on). Highly connected agents will naturally form subgroups corresponding to biologically relevant categories as exemplified by the Congenital Hepatic Fibrosis [58] gene network (Figure 4).

### Topic: Interactive Collaborative Unit

On their profile pages, users can create groups of agents (called Topics). Each group centralizes information around a research project and links to agents collaborating with it (Multimedia Appendix 4), thus presenting a subjective view of biological information from a given research perspective.

This allows MyGeneFriends to display a personalized news feed, providing a technological watch of bibliographical and public database updates related to gene and disease friends that collaborate in the user’s Topics. News items include various subjects such as new or lost friendships between diseases and genes, updated symbols, synonyms, descriptions, new or lost GO or HPO annotations, protein sequence updates, or presence in a new publication.

Several tools are provided for the analysis of Topic related agents. The network visualization facilitates the evaluation of the heterogeneity of the Topic’s content (Multimedia Appendix 5), and the identification of highly linked subgroups of agents and relationships between these groups. The timeline visualization (Figure 3) places the Topic in a global research perspective, presenting the annual evolution of the number of publications associated with the genes in a Topic.

Finally, in addition to serving as a basis for friendships and publications suggestions (see Methods), information mined from Topics allows the enhancement of the reading experience of an agent’s descriptions and publication abstracts by automatically highlighting the keywords most representative of the user’s interests (Multimedia Appendix 6).

## Discussion

### Principal Findings

By leveraging conventions and practices used in popular social networks, MyGeneFriends aims to challenge the way we interact with data by providing a first step toward a system where biological entities such as genes and genetic diseases are no longer passive concepts, but are instead proactive agents of the research process, helping and collaborating with human counterparts.

In mainstream social networks, humans can create a representation of themselves in the form of a profile, then interact with the network by writing posts, adding commentaries or likes, making new friends, and sharing and spreading information. To transpose this concept to MyGeneFriends, we had to create a network that could reflect current research efforts in genetics and medicine. To populate the network, we focused on human genetic diseases because of their broad interest, and their more direct links to genes and genomic variations compared with infectious diseases or cancer. With humans and human genetic diseases selected, the choice of the third agent was obvious as many publications and bioinformatics resources structure their information in a gene-centric manner. To interconnect the network, we adopted two of the main characteristics of real-world friendships: commonality (common friends, qualities, and interactions), and group membership (family, coworkers, and hobbies).

Compared with existing Web services, MyGeneFriends can (1) leverage user behavior to provide personalized profiles and news feeds, given each user’s specific research interests; and (2) consider user behavior as valid biological information integrated in the biological data network to be mined and influencing the discovery of connections between genes and diseases.

### Conclusions

The development of MyGeneFriends lies at the frontier between bioinformatics and the emerging science of human-data interaction, and in the future, we plan to extend the functionalities in both areas. First, genes from other model species (mouse, zebrafish, etc) will be added and connected by friendship links based on orthology. Additional friendships will be incorporated to provide a regulatory context such as friendships based on transcription factors or miRNA. Second, we believe that while humans remain special agents in this first version of MyGeneFriends, in the future the three agents will interact on the same level, with more independent and proactive genes and diseases. Research will be facilitated by better communication between different agents, with each agent able to produce and transmit new, relevant data and knowledge. A gene could, for example, find itself linked to a new disease or ask to be sequenced by his friend, the sequencer. With this increased autonomy of nonhuman agents and an independent flow of information, the role of the human in the network must clearly evolve. This evolution can be viewed either as a danger or as a source of new collaborations and opportunities.

## Appendix

Multimedia Appendix 1 MyGeneFriends relies on multiple servers, scripts, and databases. Jenkins is used to periodically execute python integration scripts, which maintain the MyGeneFriends database up to date. A backup copy of the database is created daily, and the publications table is synchronized daily with an Elasticsearch server. The MyGeneFriends website is built using the Play framework and calls an API server built with Flask to execute command line programs and download publications into the local database. Bug reports are sent to the YouTrack server.

Multimedia Appendix 2 Diseases from different, contradictory data sources are merged using two rules. First, disease A is merged with disease B if and only if B has a single link to disease A, and no other diseases have a single link to A. Second, if a disease is linked to several other diseases, a disease group called Metadisease is created.

Multimedia Appendix 3 The “differential effect on proteins” view for variants on the “Bardet-biedl syndrome 6” disease profile (a) shows that while variants (here rs74315398, rs28937875, rs587777827, rs74315399, rs74315397, rs74315396) linked to this disease affect the coding DNA sequence (CDS) of 2 transcripts of the gene MKKS, one protein coding transcript (green rectangle on Ensembl Genome Browser [b], and 1 of 3 labels in the view [a]) is not affected in the CDS.

Multimedia Appendix 4 A Topic groups genes, diseases, and humans collaborating on a shared research interest. When a human becomes friends with a new gene or disease, it is added to the active Topic. Human collaborators see all protected friends and annotations related to the Topic.

Multimedia Appendix 5 Network visualization of agents related to a Topic (here a set of Bardet-Biedl syndrome related genes on the left and a set of muscular fiber related genes on the right) help to understand how many potentially different research interests a Topic contains. The network displays highly connected agents automatically grouped together. Purple links represent STRING based relationships, green links are based on evolutionary profile similarity, red links indicate shared public human friends, and grey links shared disease friends.

Multimedia Appendix 6 Keywords most related to agents from active Topics are highlighted in (1) publication abstracts, and (2) descriptions on agent profiles. This helps to quickly identify paragraphs that may interest the reader.

## Abbreviations

CDS: coding DNA sequence
EFO: experimental factor ontology
FSS: functional semantic similarity
FTP: file transfer protocol
GEO: gene expression omnibus
GO: gene ontology
HPO: human phenotype ontology
IC: information content
IDF: inverse document frequency
MeSH: MEdical Subject Headings
NCBI: National Center for Biotechnology Information
NLTK: natural language toolkit
OMIM: Online Mendelian Inheritance in Man
ORM: object-relational mapping
PDF: portable document format
REST: representational state transfer
STRING: search tool for recurring instances of neighboring genes
TF: term frequency
UCSC: University of California, Santa Cruz
UTR: untranslated region
VCF: variant call format
VEP: variant effect predictor
