# Supplementary figures and images for: MyGeneFriends: A Social Network Linking Genes, Genetic Diseases, and Researchers
Source: J Med Internet Res. 2017 Jun 16;19(6):e212. doi: 10.2196/jmir.6676 (PMC5493784; doi:10.2196/jmir.6676)

## Slide 1
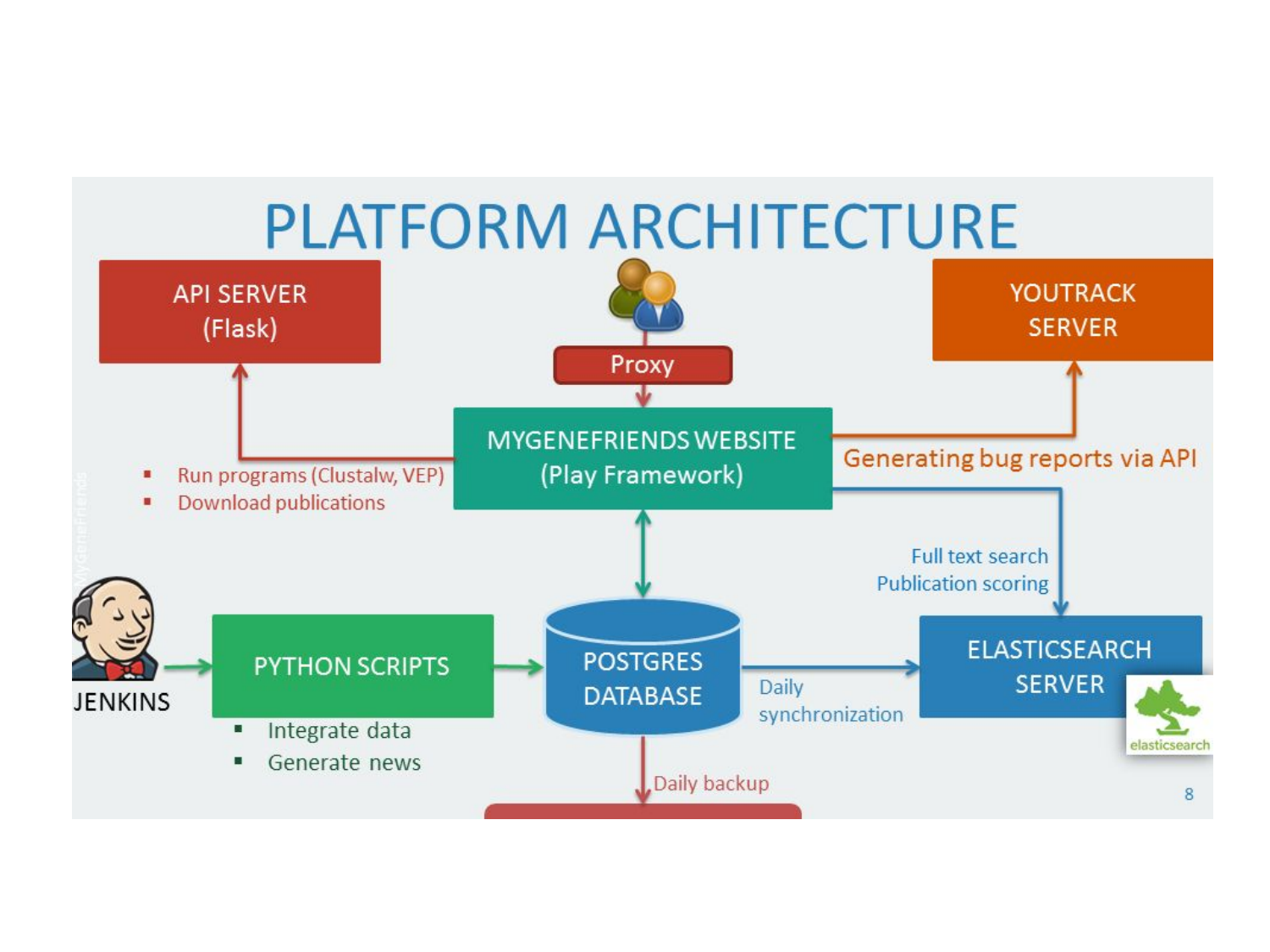

Supplement: Multimedia Appendix 1 [file jmir_v19i6e212_app1.pptx]

## Slide 1
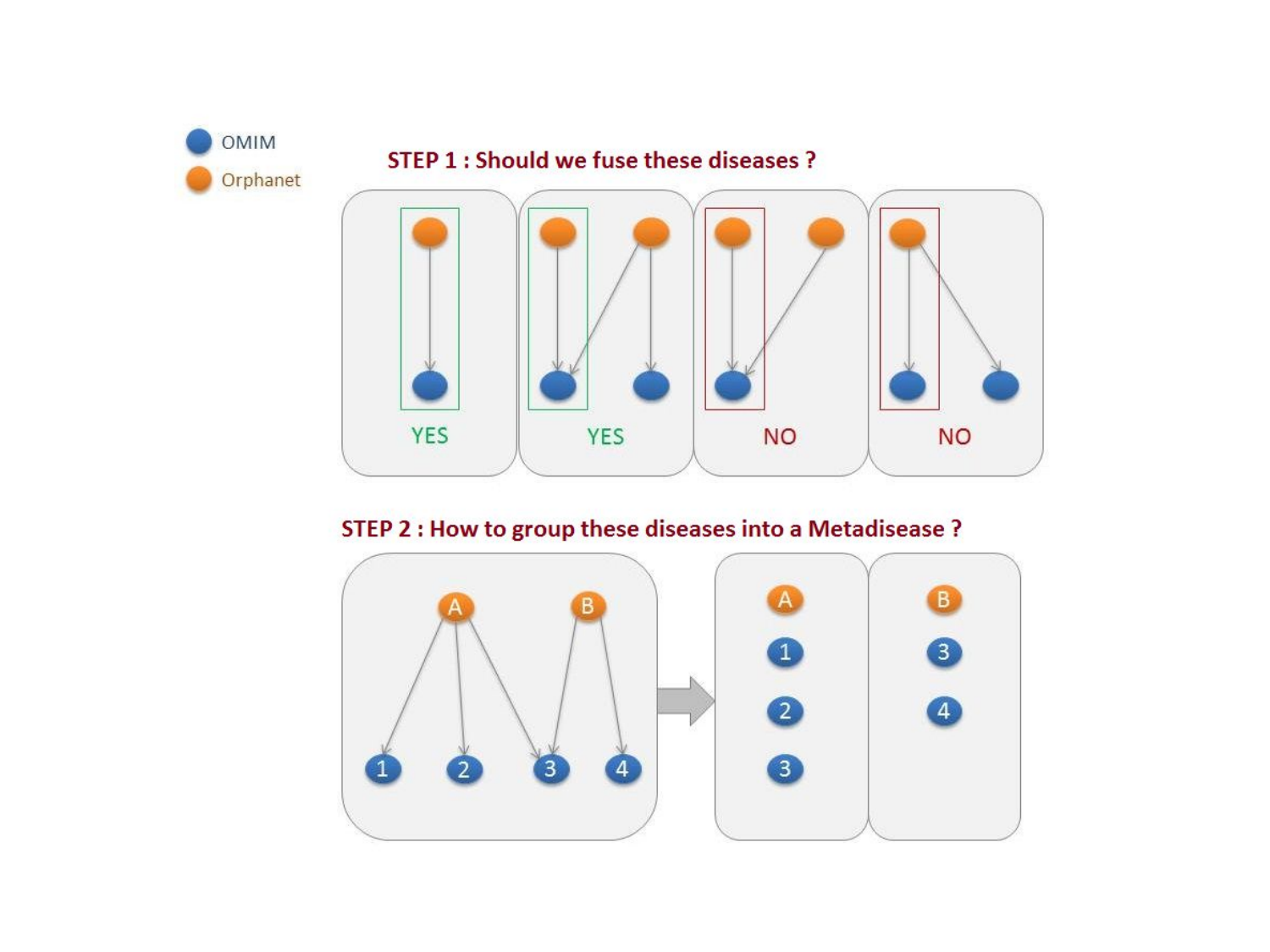

Supplement: Multimedia Appendix 2 [file jmir_v19i6e212_app2.pptx]

## Slide 1
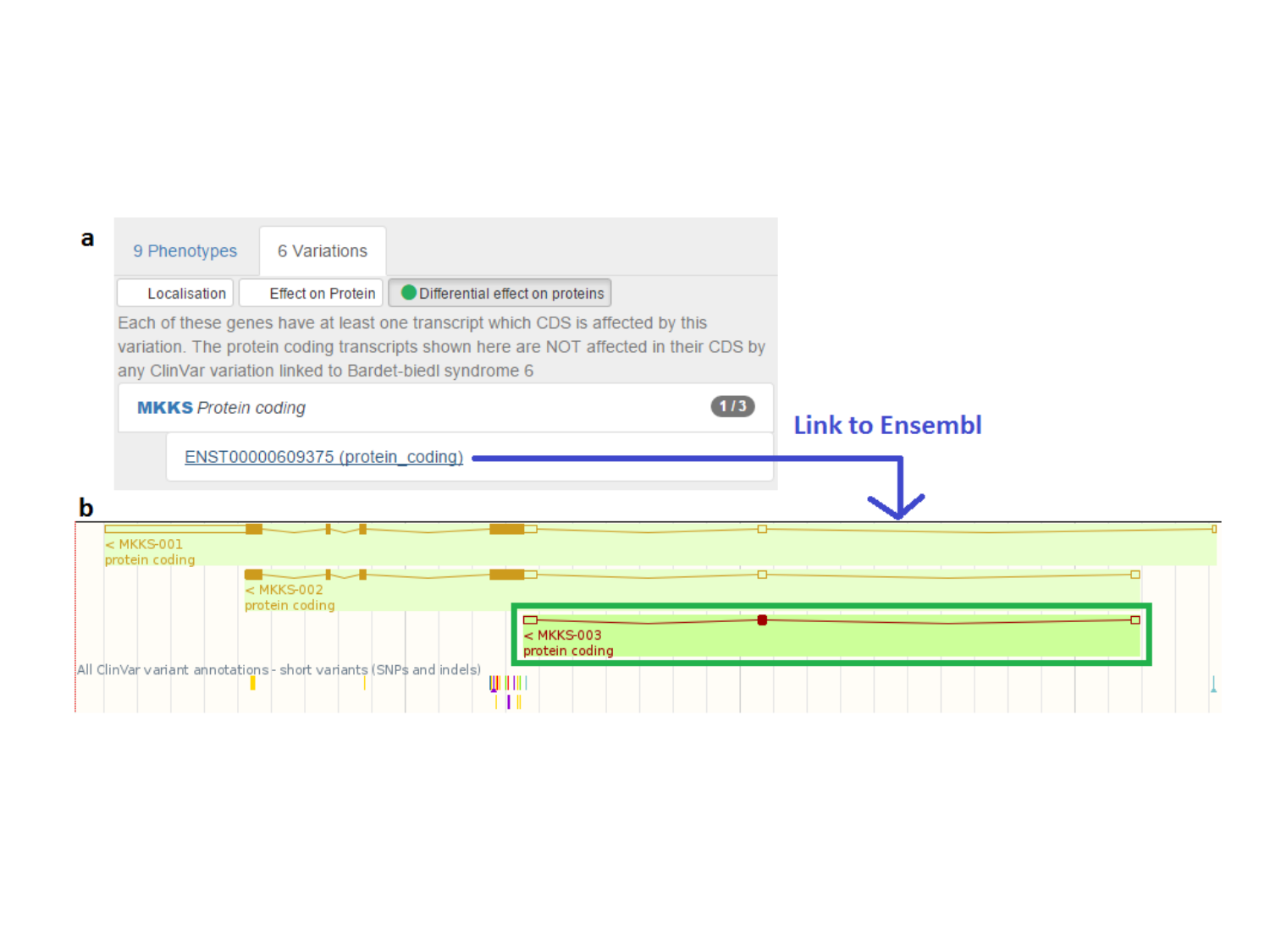

Supplement: Multimedia Appendix 3 [file jmir_v19i6e212_app3.pptx]

## Slide 1
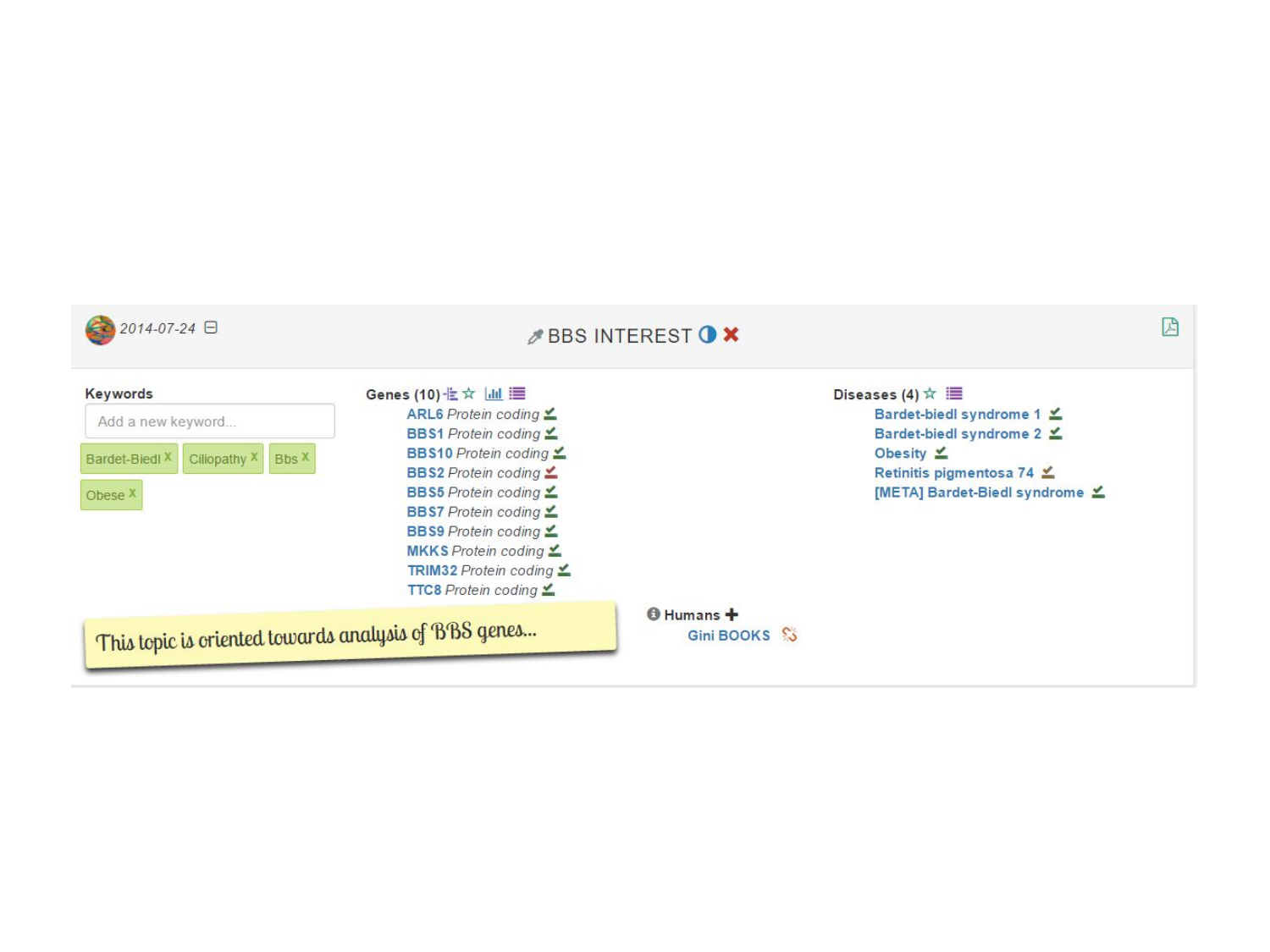

Supplement: Multimedia Appendix 4 [file jmir_v19i6e212_app4.pptx]

## Slide 1
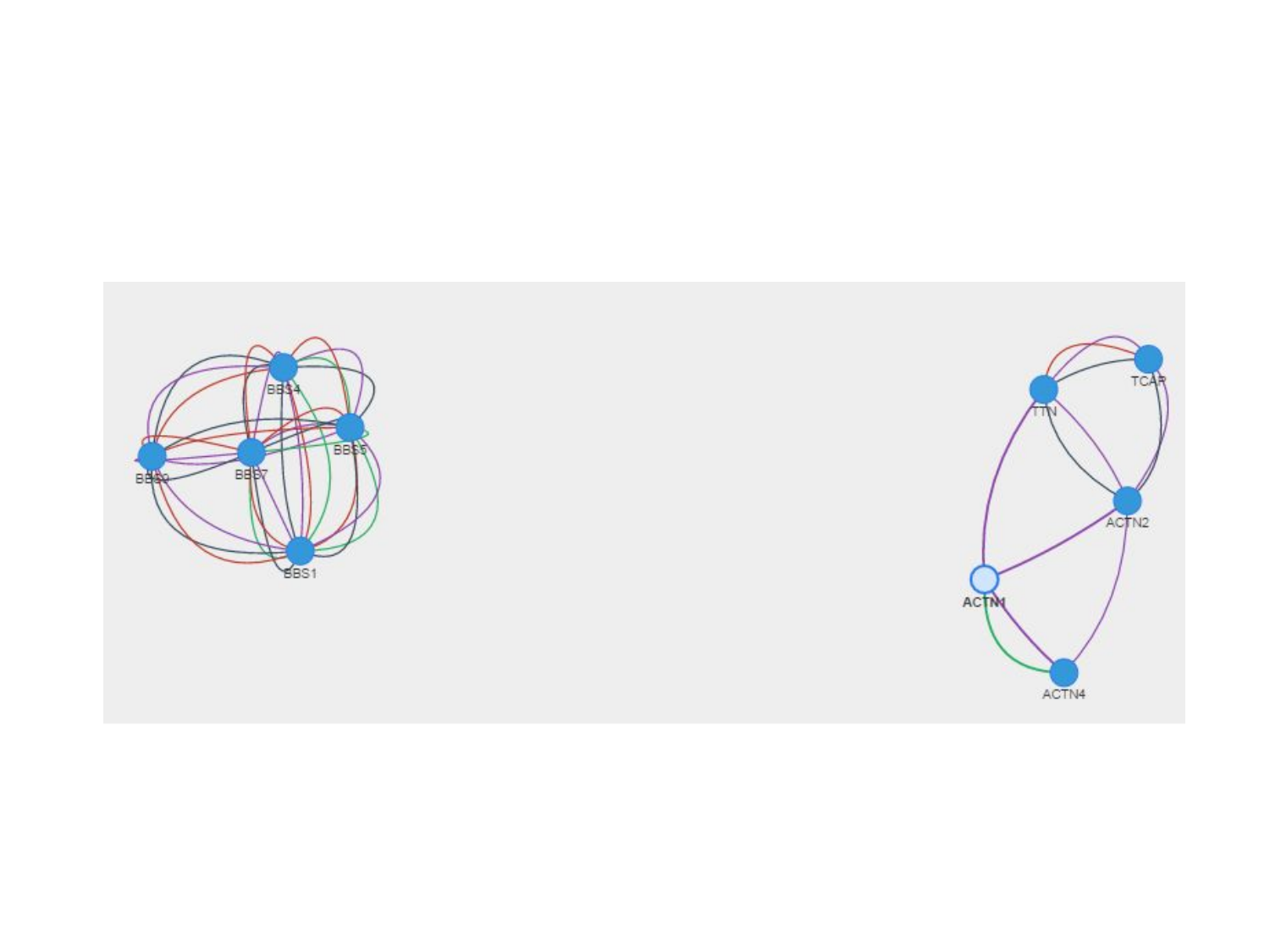

Supplement: Multimedia Appendix 5 [file jmir_v19i6e212_app5.pptx]

## Slide 1
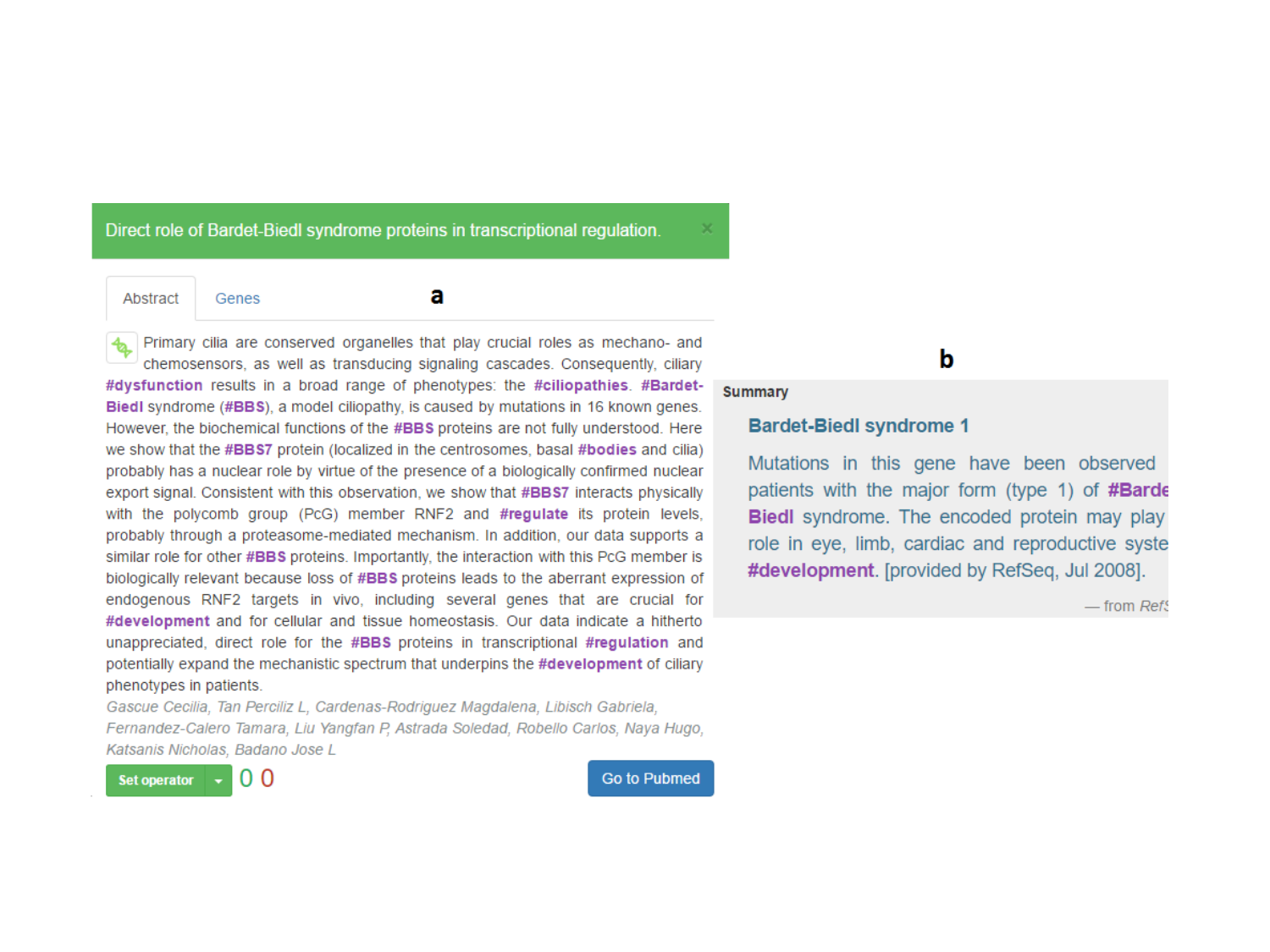

Supplement: Multimedia Appendix 6 [file jmir_v19i6e212_app6.pptx]
